# Simultaneous Exposure to *Angiostrongylus vasorum* and Vector-Borne Pathogens in Dogs from Italy

**DOI:** 10.3390/pathogens10091200

**Published:** 2021-09-15

**Authors:** Simone Morelli, Francesca Gori, Mariasole Colombo, Donato Traversa, Giulia Sarrocco, Giulia Simonato, Chiara Nespeca, Angela Di Cesare, Antonio Frangipane di Regalbono, Fabrizia Veronesi, Ilaria Russi, Manuela Schnyder

**Affiliations:** 1 Faculty of Veterinary Medicine, University of Teramo, 64100 Teramo, Italy; mcolombo@unite.it (M.C.); dtraversa@unite.it (D.T.); giulia.sarrocco@studenti.unite.it (G.S.); chiara.nespeca@studenti.unite.it (C.N.); adicesare@unite.it (A.D.C.); ilaria.russi.03@gmail.com (I.R.); 2 Institute of Parasitology, Vetsuisse-Faculty, University of Zurich, 8057 Zurich, Switzerland; francesca.gori@uzh.ch (F.G.); Manuela.schnyder@uzh.ch (M.S.); 3 Department of Animal Medicine, Production and Health, University of Padua, 35020 Lengaro, Italy; giulia.simonato@unipd.it (G.S.); antonio.frangipane@unipd.it (A.F.d.R.); 4 Department of Veterinary Medicine, University of Perugia, 06123 Perugia, Italy; fabrizia.veronesi@unipg.it

**Keywords:** *Angiostrongylus vasorum*, antigen, antibodies, vector-borne diseases

## Abstract

Several drivers have recently fostered the expansion of *Angiostrongylus vasorum* throughout Europe, where Vector-Borne Pathogens (VBPs) are also spreading. However, the level of simultaneous risk of infection is still unknown in canine populations. This study evaluated the simultaneous exposure to *A. vasorum* and major canine VBPs in dogs of Italy. Sera of 294 dogs were subjected to two ELISAs, detecting *A. vasorum* circulating antigens and antibodies against the parasite, and to the following assays: (i) SNAP® 4DX (IDEXX Laboratories Inc.) detecting *Dirofilaria immitis* antigens, and antibodies vs. *Borrelia burgdorferi*, *Anaplasma* spp. and *Ehrlichia* spp. and (ii) IFAT for the detection of antibodies vs. *Leishmania infantum*, *Babesia canis* and *Rickettsia conorii*. Twenty-two (7.5%, CI: 4.8–11.1%) and six (2%, CI: 0.7–4.4%) dogs scored positive for circulating *A. vasorum* antibodies and antigens, respectively. Seventeen dogs (5.8%, CI: 3.4–9.1%) were positive for *A. vasorum* antibodies + at least one VBP, three (1%, CI: 0.2–3%) for *A. vasorum* antigen + at least one VBP, while one dog (0.3%, CI: 0.01–1.88%) was positive for *A. vasorum* antigen + *A. vasorum* antibodies + *B. canis* antibodies. These results show that dogs living in different regions of Italy are at risk of simultaneous infections with both *A. vasorum* and VBPs. Despite the same scenario being likely in other countries of Europe, the current knowledge is scant. Therefore, further studies are warranted to amplify current epizootiological information and to understand whether control programs should be improved.

## 1. Introduction

Over the last decade, nematodes of the genus *Angiostrongylus* have changed their distribution in Europe, with the spread of *Angiostrongylus vasorum* in canids [1,2], and new or unexpected findings of other species like *Angiostrongylus cantonensis*, *Angiostrongylus chabaudi* and *Angiostrongylus daskalovi* in different animal hosts [3,4,5]. Among them, *A. vasorum* is now regarded as a primary parasite of dogs throughout European regions, e.g., Iberian Peninsula, Mediterranean Basin, and northern, central, and eastern Countries [6,7,8,9].

Vector-Borne Diseases (VBDs) are also expanding in many territories, where they are a threat to animal and human health [10,11,12,13,14]. Dogs are frequently at risk of VBDs caused by non-parasitic (e.g., bacteria, viruses) and parasitic (e.g., protozoa) Vector-Borne pathogens (VBPs) transmitted by ticks and flying insects [10,12,15,16]. In Europe, the most important and distributed tick-borne bacteria are *Ehrlichia canis*, *Anaplasma platys*, *Anaplasma phagocitophylum*, *Rickettsia conorii* and *Borrelia* spp. [17,18,19,20]. The nematode *Dirofilaria immitis* transmitted by mosquitoes, and the protozoans *Leishmania infantum* and *Babesia* spp. transmitted by sandflies and ticks, respectively, are the preeminent canine vector-borne parasites [21,22,23]. Many of these pathogens may also infect other animals, e.g., cats, and zoonotic VBPs shared by dogs and cats increase chances of interspecific transmission and of human infections [16,24,25,26,27,28,29]. 

Extrinsic and intrinsic drivers, e.g., global warming, travelling of pets with their owners, relocation and growth of human and animal population, and anthropization of wildlife habitats, may increase the distribution of pathogens transmitted by invertebrates and/or with an indirect biological cycle in both enzootic and free areas [15,29,30,31,32]. For instance, climate change has an impact on the spatial–temporal distribution of gastropod-borne nematodes and VBPs via its influence on the life cycle, survival, and reproduction rates of invertebrates and transmitted pathogens [33,34,35]. Increasing temperatures, changes in precipitations, greater climate variability, extreme weather events may favor the emergence of VBPs and of gastropod-borne nematodes in different ways. For instance, temperate areas becoming warmer are more suitable for vectors. This favors the introduction and establishment of new pathogens and vectors in previously free areas [33].

Wild animals serve as a source of infection for invertebrates and populations of domestic animals, especially if conurbation favors bridging infections [2,35,36].

In this scenario, many European regions present a suitable environment for the circulation and spread of diseases transmitted by invertebrates and overlapping factors spur the emergence of canine angiostrongylosis and VBDs [32,34,37,38,39]. Accordingly, Italy is a major epizootiological hub for angiostrongylosis and VBPs, which are now enzootic throughout the Country in both domestic and wild animals, and dogs may suffer from one or more of these diseases at the same time [25,39,40,41,42,43]. 

*Angiostrongylus vasorum* has been found in Italian territories where it was considered unexpected (e.g., Northern regions) and it may occur simultaneously with *D. immitis* even where the monthly use of macrocyclic lactones would be able to prevent both diseases [42,43,44,45,46]. A recent study has proven that canine populations living in different regions of Italy are exposed to the most important VBPs despite the use of preventative treatments [39]. These findings indicate that continuous surveillance on local epizootiological scenarios is necessary for a constant refinement of control programs. Accordingly, the present survey investigated the simultaneous exposure of privately owned dogs living in different regions of Italy to *A. vasorum* and major VBPs.

## 2. Results

### 2.1. Angiostrongylus vasorum

Of the 294 dogs examined, 27 dogs (9.2%, 95% Confidence Interval, CI: 6.1–13.1%) were exposed to *A. vasorum*, i.e., Ab or Ag of *A. vasorum* were detected in 22 (7.5%, CI: 4.8–11.1%) and 6 (2%, CI: 0.7–4.4%) dogs, respectively, while 1 dog was positive for both Ab and Ag. Four (1.4%, CI: 0.3–3.4%) and 2 (0.7%, CI: 0.08–2.4%) dogs were positive for *A. vasorum* Ab or Ag only, respectively, while the other 21 were simultaneously positive also for other VBPs (Table 1—see Section 2.2.). Seventeen (5.8%; CI: 3.4–9.1%) positive dogs were female, while 10 (3.4%, CI: 1.6–6.1%) were male. Four dogs (1.4%, CI: 0.3–3.4%) positive to *A. vasorum* Ab lived mostly indoors, while all the other 23 dogs (7.8%. CI: 5–11.5%) lived permanently outdoors. The median age of dogs that seroreacted for *A. vasorum* was 36 months.

### 2.2. Vector-Borne Pathogens

Regardless of the positivity to *A. vasorum*, 162 (55.1%, CI: 49.2–60.8%) dogs were seropositive for at least one VBP, while 40 (13.6%, CI: 9.9–18.1%) scored positive to two or more VBPs (Table 1). Ab vs. *Rickettsia conorii* were found in 136 dogs (46.3%, CI: 40.5–52.1%), while 25 (8.5%, CI: 5.6-12.3%) and 18 (6.1%, CI: 3.7–9.5%) dogs seroreacted for *Babesia canis* and *L. infantum,* respectively. Twenty-two (7.5%, CI: 4.8–11.1%) dogs were seropositive for *Anaplasma* spp., while 4 (1.4%, CI: 0.3–3.4%) and 1 (0.3%, CI: 0.01–1.88%) dogs had antibodies against *B. burgdorferi* and *Ehrlichia* spp. *D. immitis* antigens were detected in 5 (1.7%, CI: 0.6–3.9%) dogs. Positivity rates for VBPs for each macro-area are detailed in Table 1. Of the dogs positive to at least one VBP, 86 (29.3%, CI: 24.1–34.8%) dogs were female, while 81 (27.6%, CI: 22.5–33.0%) were male: 117 (39.8%, CI: 34.2–45.6%) lived outdoors, while 50 (17.0%, CI: 12.9–21.8%) lived indoors. The median age of dogs that tested positive for one or more VBPs was 54 months.

### 2.3. Simultaneous Exposure

Twenty-one (7.1%, CI: 4.5–10.7%) dogs tested simultaneously positive to both *A. vasorum* (Ag or Ab) and at least one VBP. Of them, 17 (5.8%, CI: 3.4–9.1%) were positive for *A. vasorum* Ab + at least one VBP, 3 (1%, CI: 0.2–3%) were positive for *A. vasorum* Ag + at least one VBP, while 1 dog (0.3%, CI: 0.01–1.88%) was positive for both *A. vasorum* Ag and Ab, and for *B. canis* Ab.

Detailed information on the different combinations of seropositivity to the tested pathogens are reported in Table 1 and Table 2, respectively.

## 3. Discussion

The present results show that dogs living in Italy can be simultaneously exposed to *A. vasorum* and several VBPs, with different positivity rates according to the investigated geographic areas. 

Exposure to *A. vasorum* was herein found in all the studied macro-areas. Thus far, this metastrongyloid has been rarely recorded outside Central and Southern Italy, in particular seldom in Northern-Western territories [41]. Nevertheless, a relatively high proportion of dogs of the present study tested seropositive in Site A. This and other recent findings [42] indicate that *A. vasorum* has currently expanded its distribution range and confirm that also dogs living in North-Eastern regions of Italy are at risk of canine angiostrongylosis. A past study investigated the occurrence of *A. vasorum* in dogs from a small region (Liguria) of North-Western Italy, showing ~1 and ~3% positivity to Ag and AB, respectively [47]. The present data indicate that *A. vasorum* can occur in Northern Italy more frequently than previously recorded, although the nematode remains more distributed in Central Italy rather than in the North of the country [41]. This difference is likely due to the chemoprevention for *D. immitis* using macrocyclic lactones (i.e., milbemycin oxime and moxidectin) that are active also against *A. vasorum*, routinely performed in Northern Italy [44]. No positive dogs were detected in Giglio Island of Site B, most probably attributable to the absence of foxes, the natural reservoirs of *A. vasorum*, that are not present in minor Italian islands [48]. However, a future introduction of the parasite in such spots, e.g., via dogs travelling with their owners in touristic areas, is not unlikely, as metastrongyloids can perpetuate their lifecycle in absence of their natural wild reservoirs [49].

As in previous studies, the number of dogs with detectable *A. vasorum* Ab was here higher if compared to the number of dogs showing circulating Ag [8,47,50]. Many reasons can account for this discrepancy, e.g., the inclusion of dogs (i) sampled during prepatency when Ag is not detectable yet, (ii) after a natural clearance of the infection (iii) after antiparasitic treatment, based on longer persistence of Ab [51,52]. Moreover, individual variations in the production of Ab against *A. vasorum* (i.e., the production of antibodies may vary individually and some dogs can even remain antibody negative) may further reduce the number of detected positive dogs [53,54]. 

*Dirofilaria immitis* was here found in all study sites and most of the positive dogs lived in central regions, which are now regarded as stably enzootic. Seropositivity to *L. infantum* was detected in dogs living on the island of Giglio and in Umbria/Abruzzo/Marche, but not in Friuli Venezia Giulia region (i.e., Site B, the easternmost study site). The lack of positive dogs may not reflect a true absence in this latter region, as competent vectors are already established in this area [55]. The presented results and recent analysis [56] ultimately prove that, to date, all Italian regions should be considered potentially enzootic for *D. immitis* and *L. infantum* and corroborate the changing epizootiological scenario of dog dirofilariosis in Italy, following an expansion from Northern areas southwards [56,57]. Hence, worthy of note is the establishment of hyperendemic foci of dirofilariosis in the extreme South of Italy, and vice versa for leishmaniosis [58,59,60,61]. 

The seropositivity of the here studied dog populations to tick borne pathogens fits with past and most recent surveys carried out in Italy and confirm that dogs are exposed to a multitude of pathogens throughout the country [17,39,62,63] (Table 3). The level of exposure to given pathogens in each geographic area is in accordance with the biology of their vectors. For instance, *R. conorii* and *Anaplasma* spp. are transmitted by ticks (e.g., *Rhipicephalus sanguineus* and *Ixodes ricinus*) which are widespread and active throughout the year [64,65,66]. Conversely, the main vector of *B. canis* (*Dermacentor* spp.) is little distributed in northern territories and limited to a short season [65,67]. On the other hand, the positivity values in central and southern areas could be due to possible cross-reactions with other *Babesia* species, e.g., *Babesia vogeli* or others, transmitted by widely spread ticks like *R. sanguineus* [63,68,69]. Analogously, the occurrence of *B. burgdorferi* in northern Italy is explained by the wide distribution of its major vector *I. ricinus* in northern areas, with forested environments and suitable climate [65].

Worthy of note is the positivity to *R. conorii*, as dogs are reliable epidemiological sentinels for monitoring potential risk of human exposure to spotted fever group rickettsioses. Regardless of the potential of cross-reactions with other *Rickettsia* species, *R. conorii* is the most frequent affecting dogs in the Mediterranean basin, and the present data indicate a high exposure to infected tick arthropods, which act as vectors of these pathogens [63].

Knowledge on the simultaneous risk for angiostrongylosis and VBDs in dog populations of Europe is very poor, as studies have thus far been conducted only in a few countries, e.g., Portugal [37] and Bulgaria [70,71]. In the latter studies, cases of co-exposure /co-infection by *A. vasorum* and VBPs have not been found [37]. In Italy, a similar study was carried out a few years ago in kenneled dogs of central Italy, though limited to *L. infantum*, *A. vasorum* and *Dirofilaria* spp. (and other endoparasites) [106]. Unfortunately, a comparison with the number of dogs co-infected by VBPs and *A. vasorum* is not possible, as this information is not shown in ref. [106]. Further differences in the diagnostic methods and/or protocols, e.g., the use of Baermann test for *A. vasorum* vs. antigen detection by ELISA or rapid assay [52], of higher screening dilution for *L. infantum* and of a different commercial kit for *D. immitis* antigen detection, impair any comparison with the present results.

This study has confirmed once again the applicability of serological methods in large-numbers epizootiological studies and investigations on pet metastrongyloid angiostrongylids [47,50,51,132,133,134,135]. The usefulness of serology should be considered in planning future studies relying on standardized methods towards comparable results from different regions and settings.

Many European countries are enzootic for *A. vasorum* and VBPs (Table 3). Hence, the simultaneous exposure to these pathogens is a realistic threat even where this has not been verified by purposed studies, especially if one considers that these pathogens are spreading in both enzootic and previously free regions [35,36,136,137].

As the scenario of the distribution of canine parasites and VBDs is in continuous evolution, further large-scale studies are warranted for more comprehensive knowledge on the epizootiological risks in European territories, and to understand if and how the routine use of broad-spectrum endo-ectoparasiticides needs a refinement based on local settings. The use of ectoparasiticides and/or repellents is the most effective strategy to minimize the risk of diseases transmitted by arthropod vectors, while dog angiostrongylosis and dirofilariosis can be prevented by the monthly administration of macrocyclic lactones like milbemycin oxime and moxidectin [138,139,140,141]. Although cases of LOE/ML-resistant strain of heartworm are known to occur in North America, this is not the case in Europe [142,143]. Several broad-spectrum formulations are available on the market to protect dogs and those containing an isoxazoline and either or milbemycin oxime or moxidectin may be used for the prevention of ticks, *D. immitis* and *A. vasorum* infections. Although isoxazolines do not have a repellent activity, their fast onset of action has the potential to reduce the risk of disease transmission of tick-borne pathogens that are not immediately transmitted to the host, such as *Babesia* or *Borrelia* [144]. Repellents (e.g., synthetic pyrethroids) have a fast onset of action on many insects and ticks, and they are also irritant or repulsive for many ectoparasites [144,145]. Their use is thus particularly important for the prevention of *L. infantum* [146,147] and of pathogens that are passed within a few hours or immediately after the tick’s bite [148]. In fact, in recent years, the use of systemic isoxazolines to reduce the transmission of leishmaniosis or human VBDs has been evaluated [149,150,151]. Further studies in this field are still necessary and, to date, the combination of repellency and parasiticidal activity is still the best approach to prevent pathogen transmission by arthropods. Therefore, an adequate preventative measure should be put in place on a case-by-case basis considering the lifestyle of the dog, the geographical distribution of vectors and of the related transmitted diseases. 

In conclusion, canine populations of Italy and in parts of other European countries are at factual risk of angiostrongylosis and VBDs at the same time. A standardization of the diagnostic techniques used in future epizootiological surveys is here advocated, along with the establishment of international monitoring tools with real-time mapping of positive animals in determined areas, e.g., as available in the USA via the CAPC (capcvet.org).

## 4. Materials and Methods

Overall, 294 privately owned dogs from North-Eastern (Friuli Venezia-Giulia and Veneto = site A), Central-Western (Giglio Island of Tuscany, Latium = Site B) and Central-Eastern (Umbria, Abruzzo, Marche = Site C) Italy were enrolled in the study, i.e., 67, 82 and 145 in each site, respectively. All the dogs were apparently healthy at the clinical examination. Overall, 150 dogs were female, 144 were male and the median age was 48 months. Of the 294 dogs included, 187 lived permanently outdoors, while 107 lived mostly indoors.

Dogs were enrolled during routine medical checks by local veterinarians and all dog owners signed a written consent form before sampling. All sera samples were subjected to two ELISAs detecting *A. vasorum* circulating antigen (Ag) (Sensitivity 95.7%, Specificity 94.0%) and specific antibodies (Ab) (Sensitivity 81.0%, Specificity 98.8%) against the parasite [54,152]. The optical density (OD) threshold was determined for both Ag (A_405_ nm = 0.153) and Ab test (A_405_ nm = 0.266) using the mean OD value plus three standard deviations of 291 (Ag) and 244 (Ab) samples. All dogs were tested also for VBPs using (i) SNAP® 4DX (IDEXX Laboratories, Inc., Westbrook, ME, USA) detecting *D. immitis* Ag (Sensitivity 99%, Specificity 99.3%) and Ab vs. *B. burgdorferi* (Sensitivity 94.1%, Specificity 96.2%), *Anaplasma* spp. (Sensitivity 90.3%, Specificity 94.3%) and *Ehrlichia* spp. (Sensitivity 97.1%, Specificity 95.3%) and (ii) IFAT for Ab vs. *L. infantum* (MegaFLUO Leish-Megacor Diagnostik GmbH) (Sensitivity 96.9%, Specificity 98.7%), *B. canis* (MegaFLUO BABESIA *canis*-Megacor Diagnostik GmbH) and *R. conorii* (MegaFLUO RICKETTSIA *conorii*-Megacor Diagnostik GmbH) using screening dilutions of 1:100, 1:160 and 1:64, respectively.

## Figures and Tables

**Table 1:** Results of serological examinations (SNAP 4DX rapid test; Immunofluorescence Antibody Test, IFAT; ELISA): number/total (n/tot) and percentage (%) of dogs positive for different pathogens in Italy ^§^.

	SNAP 4DX				IFAT					ELISA *Av*			
Site	*An* n/tot (%)	*Eh* n/tot (%)	*Bb* n/tot (%)	*Di* n/tot (%)	*Li* n/tot (%)	*Rc* n/tot (%)	*Bc* n/tot (%)	Mixed VBP ^a^ n/tot (%)	Total VBP ^b^ n/tot (%)	Ag n/tot (%)	Ab n/tot (%)	Total *Av* ^d^ n/tot (%)	Mixed VBP + *Av* ^c^ n/tot (%)
**A**	8/67 (11.9)	- -	4/67 (6)	2/6 (3)	1/6 (1.6)	41/67 (61.2)	- -	10/67 (14.9)	45/67 (67.2)	- -	5/67 (7.5)	5/67 (7.5)	4/67 (6)
**B**	9/82 (11)	- -	- -	1/82 (1.2)	3/82 (3.7)	39/82 (47.6)	18/82 (22)	18/82 (22)	48/82 (58.5)	- -	8/82 (9.8)	8/82 (9.8)	8/82 (9.8)
**C**	5/145 (3.4)	- -	- -	2/145 (1.4)	14/145 (9.7)	54/145 (37.2)	7/145 (4.8)	12/145 (8.3)	69/145 (47.6)	6/145 (4.1)	9/145 (6.2)	14/145 (9.7)	9/145 (6.2)
**Total**	22/294 (7.5)	1/294 (0.3)	4/294 (1.4)	5/294 (1.7)	18/294 (6.1)	136/294 (46.3)	25/294 (8.5)	40/294 (13.6)	162/294 (55.1)	6/294 (2)	22/294 (7.5)	27/294 (9.2)	21/294 (7.1)

*An*: *Anaplasma* spp.; *Eh*: *Ehrlichia* spp.; *Bb*: *Borrelia burgdorferi*; *Di*: *Dirofilaria immitis*; *Li*: *Leishmania infantum*; *Rc*: *Rickettsia conorii*; *Bc*: *Babesia canis*; *Av*: *Angiostrongylus vasorum*; *Ag*: Antigen; Ab: Antibodies; ^a^ Dogs that tested positive for two or more pathogens investigated in this study; ^b^ Dogs positive for at least one VBP; ^c^ Dogs positive to at least one VBP and to *Angiostrongylus vasorum* (*Ag* or *Ab*) simultaneously; ^d^ Dogs positive for *Angiostrongylus vasorum* antigens or antibodies; ^§^ Sites of North-Eastern (i.e., Site A: Veneto and Friuli-Venezia Giulia), Central-Western (i.e., Site B: Giglio Island, Tuscany and Latium) and Central-Eastern (i.e., Site C: Umbria, Abruzzo, Marche) macroareas of Italy.

**Table 2:** Number (n) and percentage (%) of dogs seropositive for *Angiostrongylus vasorum* antigens (Ag) and/or antibodies (Ab) and combined exposure to VBPs in the present study.

Simultaneous Exposure	n (%)
*Angiostrongylus vasorum* Ab + *Rickettsia conorii*	9 (3.0)
*Angiostrongylus vasorum* Ab + *Leishmania infantum*	1 (0.3)
*Angiostrongylus vasorum* Ab + *Anaplasma* spp. + *Rickettsia conorii*	3 (1.0)
*Angiostrongylus vasorum* Ab + *Rickettsia conorii* + *Babesia canis*	2 (0.7)
*Angiostrongylus vasorum* Ab + *Anaplasma* spp. + *Borrelia burgdorferi*	1 (0.3)
*Angiostrongylus vasorum* Ab + *Leishmania infantum* + *Rickettsia conorii* + *Babesia canis*	1 (0.3)
*Angiostrongylus vasorum* Ag + *Babesia canis*	2 (0.7)
*Angiostrongylus vasorum* Ag + *Rickettsia conorii*	1 (0.3)
*Angiostrongylus vasorum* Ab + Ag + *Babesia canis*	1 (0.3)

**Table 3:** Positivity (%) of *Angiostrongylus vasorum* and Vector-Borne Pathogens in dogs of different European countries between 2006 and 2021, detected using different diagnostic techniques. *Av*: *Angiostrongylus vasorum* (Baermann test, serological detection of antigens (Ag) or antibodies (Ab)). *Li*: *Leishmania infantum* (serological detection of antibodies, PCR); *Di*: *Dirofilaria immitis* (serological detection of antigens, Knott’s test, PCR); *Eh*: *Ehrlichia* spp. (serological detection of antibodies, PCR); *Anaplasma* spp. (serological detection of antibodies, PCR); *Bo*: *Borrelia* spp. (serological detection of antibodies, PCR); *Rc*: *Rickettsia conorii* (serological detection of antibodies, PCR); *Ba*: *Babesia canis* (serological detection of antibodies, PCR). Only countries for which information on both *A. vasorum* and VBPs is available are shown in the table.

Country	*Angiostrongylus vasorum*			*Leishmania infantum*	*Dirofilaria immitis*	*Ehrlichia* spp.	*Anaplasma* spp.	*Borrelia burgdorferi*	*Rickettsia conorii*	*Babesia canis*	References
	*Baermann*	*Ag*	*Ab*								
Bulgaria		0–0.6	0	0	10.5–16.2	1.34–21	13.4–46.1	0.7–2.4		16.2	[70,71,72]
Czech Republic	0.4	4.8	3.6				3.4 *	6.5–10.3			[73,74,75]
Denmark	1.1–3.5										[76]
Finland	CR					0.3	5.3	2.9			[77,78]
France		1.8	3.5		0.2	0.3	2.7	1.1			[79,80]
Germany	0.4–7.4	0.5	2.2	5–23.5	1–1.4	0.1–15.1	1.5–43.2	4.5	0.8	0.1–11.5	[51,81,82,83,84,85,86,87]
Greece	1.1	1.6–2.0	1.4–3.0	6.5–25.2		1.5–12.5	1–6.2	0–0.1	11 *–46.5	0.5	[18,88,89,90,91,92]
Hungary		3.1	4.09	0–2.6 *	1.3 *–2.6	0.2	1.3 *–15.4	0.4		11.5–50 *	[93,94,95]
Ireland	0.5										[96]
Italy	0.4–12.6	0.8–0.9	2.3–3.8	2.5–54.1	8.9	0.4–46	0.5 *–38	0.3–1.7	40.5–72	10.3–70	[39,40,41,47,63,97,98,99,100,101,102,103,104,105,106]
Poland		1.3	1.79			0 *–1.5	8–12.3	3.8–11			[107,108,109]
Portugal	2.0	0–2.6	1.9–5	18.2–60.4 *	9.4				0.5 *–38.5		[6,37,38,110,111,112,113,114]
Serbia	CR			10.6		0–0.9	0–28.8	0 *–1.8		2.7 *–52.3	[115,116,117]
Slovakia	CR	3.1	4.4				11.7	2.8			[50,118,119,120]
Spain		0.8		5.3–30	0–6.25	0.9–16.7	1.3–19	0–6.3	42–56.4		[121,122,123,124,125,126,127,128,129,130,131]

* PCR; CR: Case Report.

## Data Availability

All study data are presented in the article.

## References

[1] Elsheikha H.M., Holmes S.A., Wright I., Morgan E.R., Lacher D.W. (2014). Recent advances in the epidemiology, clinical and diagnostic features, and control of canine cardio-pulmonary angiostrongylosis. Vet. Res..

[2] Gillis-Germitsch N., Tritten L., Hegglin D., Deplazes P., Schnyder M. (2020). Conquering Switzerland: The emergence of *Angiostrongylus vasorum* in foxes over three decades and its rapid regional increase in prevalence contrast with the stable occurrence of lungworms. Parasitology.

[3] Gherman C.M., Deak G., Matei I.A., Ionică A.M., D’Amico G., Taulescu M., Barbu-Tudoran L., Sarmaşi A., Mihalca A.D., Cozma V. (2016). A rare cardiopulmonary parasite of the European badger, *Meles meles*: First description of the larvae, ultrastructure, pathological changes and molecular identification of *Angiostrongylus daskalovi* Janchev & Genov 1988. Parasites Vectors.

[4] Paredes-Esquivel C., Sola J., Delgado-Serra S., Puig Riera M., Negre N., Miranda M.Á., Jurado-Rivera J.A. (2019). *Angiostrongylus cantonensis* in North African hedgehogs as vertebrate hosts, Mallorca, Spain, October 2018. EuroSurveillance.

[5] Di Cesare A., Morelli S., Colombo M., Simonato G., Veronesi F., Marcer F., Diakou A., D’Angelosante R., Pantchev N., Psaralexi E. (2020). Is Angiostrongylosis a Realistic Threat for Domestic Cats?. Front. Vet. Sci..

[6] Alho A.M., Schnyder M., Schaper R., Meireles J., Belo S., Deplazes P., de Carvalho L.M. (2016). Seroprevalence of circulating *Angiostrongylus vasorum* antigen and parasite-specific antibodies in dogs from Portugal. Parasitol. Res..

[7] Grandi G., Lind E.O., Schaper R., Ågren E., Schnyder M. (2017). Canine angiostrongylosis in Sweden: A nationwide seroepidemiological survey by enzyme-linked immunosorbent assays and a summary of five-year diagnostic activity (2011–2015). Acta Vet. Scand..

[8] Deak G., Gillis-Germitsch N., Ionică A.M., Mara A., Păstrav I.R., Cazan C.D., Ioniță M., Mitrea I.L., Răileanu C., Bărburaș D. (2019). The first seroepidemiological survey for *Angiostrongylus vasorum* in domestic dogs from Romania. Parasites Vectors.

[9] Tachmazidou A., Papaioannou N., Diakou A., Savvas I., Patsikas M., Stylianaki I., Morelli S., Di Cesare A., Mylonakis M.E. (2021). First report of fatal autochthonous angiostrongylosis in a dog in Greece. Vet. Parasitol. Reg. Stud. Rep..

[10] Simón F., Siles-Lucas M., Morchón R., González-Miguel J., Mellado I., Carretón E., Montoya-Alonso J.A. (2012). Human and animal dirofilariasis: The emergence of a zoonotic mosaic. Clin. Microbiol. Rev..

[11] Gharbi M., Mhadhbi M., Rejeb A., Jaouadi K., Rouatbi M., Darghouth M.A. (2015). Leishmaniosis (*Leishmania infantum* infection) in dogs. Rev. Sci. Tech. Off. Int. Epiz..

[12] Solano-Gallego L., Sainz Á., Roura X., Estrada-Peña A., Miró G. (2016). A review of canine babesiosis: The European perspective. Parasites Vectors.

[13] Barash N.R., Thomas B., Birkenheuer A.J., Breitschwerdt E.B., Lemler E., Qurollo B.A. (2019). Prevalence of *Babesia* spp. and clinical characteristics of *Babesia vulpes* infections in North American dogs. J. Vet. Intern. Med..

[14] Guo W.P., Xie G.C., Li D., Su M., Jian R., Du L.Y. (2020). Molecular detection and genetic characteristics of *Babesia gibsoni* in dogs in Shaanxi Province, China. Parasites Vectors.

[15] Beugnet F., Marié J.L. (2009). Emerging arthropod-borne diseases of companion animals in Europe. Vet. Parasitol..

[16] Otranto D., Dantas-Torres F., Breitschwerdt E.B. (2009). Managing canine vector-borne diseases of zoonotic concern: Part one. Trends Parasitol..

[17] Sainz Á., Roura X., Miró G., Estrada-Peña A., Kohn B., Harrus S., Solano-Gallego L. (2015). Guideline for veterinary practitioners on canine ehrlichiosis and anaplasmosis in Europe. Parasites Vectors.

[18] Diakou A., Di Cesare A., Morelli S., Colombo M., Halos L., Simonato G., Tamvakis A., Beugnet F., Paoletti B., Traversa D. (2019). Endoparasites and vector-borne pathogens in dogs from Greek islands: Pathogen distribution and zoonotic implications. PLoS Negl. Trop. Dis..

[19] Springer A., Glass A., Topp A.K., Strube C. (2020). Zoonotic Tick-Borne Pathogens in Temperate and Cold Regions of Europe—A Review on the Prevalence in Domestic Animals. Front. Vet. Sci..

[20] Mrljak V., Kuleš J., Mihaljević Ž., Torti M., Gotić J., Crnogaj M., Živičnjak T., Mayer I., Šmit I., Bhide M. (2017). Prevalence and Geographic Distribution of Vector-Borne Pathogens in Apparently Healthy Dogs in Croatia. Vector-Borne Zoonotic Dis..

[21] McCall J.W., Genchi C., Kramer L.H., Guerrero J., Venco L. (2008). Heartworm disease in animals and humans. Adv. Parasitol..

[22] Paltrinieri S., Solano-Gallego L., Fondati A., Lubas G., Gradoni L., Castagnaro M., Crotti A., Maroli M., Oliva G., Roura X. (2010). Guidelines for diagnosis and clinical classification of leishmaniasis in dogs. J. Am. Vet. Med. Assoc..

[23] Strobl A., Künzel F., Tichy A., Leschnik M. (2020). Complications and risk factors regarding the outcomes of canine babesiosis in Central Europe—A retrospective analysis of 240 cases. Acta Vet. Hung..

[24] Diakou A., Di Cesare A., Accettura P.M., Barros L., Iorio R., Paoletti B., Frangipane di Regalbono A., Halos L., Beugnet F., Traversa D. (2017). Intestinal parasites and vector-borne pathogens in stray and free-roaming cats living in continental and insular Greece. PLoS Negl. Trop. Dis..

[25] Morelli S., Crisi P.E., Di Cesare A., De Santis F., Barlaam A., Santoprete G., Parrinello C., Palermo S., Mancini P., Traversa D. (2019). Exposure of client-owned cats to zoonotic vector-borne pathogens: Clinic-pathological alterations and infection risk analysis. Comp. Immunol. Microbiol. Infect. Dis..

[26] Morelli S., Colombo M., Dimzas D., Barlaam A., Traversa D., Di Cesare A., Russi I., Spoletini R., Paoletti B., Diakou A. (2020). *Leishmania infantum* seroprevalence in cats from touristic areas of Italy and Greece. Front. Vet. Sci..

[27] Neves M., Lopes A.P., Martins C., Fino R., Paixão C., Damil L., Lima C., Alho A.M., Schallig H.D.F.H., Dubey J.P. (2020). Survey of *Dirofilaria immitis* antigen and antibodies to *Leishmania infantum* and *Toxoplasma gondii* in cats from Madeira Island, Portugal. Parasites Vectors.

[28] Lappin M.R., Tasker S., Roura X. (2020). Role of vector-borne pathogens in the development of fever in cats: 2. Tick- and sandfly-associated diseases. J. Feline Med. Surg..

[29] Cardoso L., Schallig H., Persichetti M.F., Pennisi M.G. (2021). New epidemiological aspects of animal leishmaniosis in Europe: The role of vertebrate hosts other than dogs. Pathogens.

[30] Otranto D., Deplazes P. (2019). Zoonotic nematodes of wild carnivores. Int. J. Parasitol. Parasites Wildl..

[31] Wright I., Jongejan F., Marcondes M., Peregrine A., Baneth G., Bourdeau P., Bowman D.D., Breitschwerdt E.B., Capelli G., Cardoso L. (2020). Parasites and vector-borne diseases disseminated by rehomed dogs. Parasites Vectors.

[32] Beugnet F., Chalvet-Monfray K. (2013). Impact of climate change in the epidemiology of vector-borne diseases in domestic carnivores. Comp. Immunol. Microbiol. Infect. Dis..

[33] Ogden N.H., Lindsay L.R. (2016). Effects of Climate and Climate Change on Vectors and Vector-Borne Diseases: Ticks are different. Trends Parasitol..

[34] Caminade C., McIntyre K.M., Jones A.E. (2019). Impact of recent and future climate change on vector-borne diseases. Ann. N. Y. Acad. Sci..

[35] Otranto D., Cantacessi C., Pfeffer M., Dantas-Torres F., Brianti E., Deplazes P., Genchi C., Guberti V., Capelli G. (2015). The role of wild canids and felids in spreading parasites to dogs and cats in Europe. Part I: Protozoa and tick-borne agents. Vet. Parasitol..

[36] Otranto D., Cantacessi C., Dantas-Torres F., Brianti E., Pfeffer M., Genchi C., Guberti V., Capelli G., Deplazes P. (2015). The role of wild canids and felids in spreading parasites to dogs and cats in Europe. Part II: Helminths and arthropods. Vet. Parasitol..

[37] Maia C., Coimbra M., Ramos C., Cristovão J.M., Cardoso L., Campino L. (2015). Serological investigation of *Leishmania infantum*, *Dirofilaria immitis* and *Angiostrongylus vasorum* in dogs from southern Portugal. Parasites Vectors.

[38] Alho A.M., Pita J., Amaro A., Amaro F., Schnyder M., Grimm F., Custódio A.C., Cardoso L., Deplazes P., de Carvalho L.M. (2016). Seroprevalence of vector-borne pathogens and molecular detection of *Borrelia afzelii* in military dogs from Portugal. Parasites Vectors.

[39] Colombo M., Morelli S., Simonato G., Di Cesare A., Veronesi F., Frangipane di Regalbono A., Grassi L., Russi I., Tiscar P.G. (2021). Exposure to major vector-borne diseases in dogs subjected to different preventative regimens in endemic areas of Italy. Pathogens.

[40] Gizzarelli M., Foglia Manzillo V., Ciuca L., Moroglione M.E., El Houda Ben Fayala N., Cringoli G., Oliva G., Rinaldi L., Maurelli M.P. (2019). Simultaneous detection of parasitic vector borne diseases: A robust cross-sectional survey in hunting, stray and sheep dogs in a Mediterranean area. Front. Vet. Sci..

[41] Traversa D., Morelli S., Cassini R., Crisi P.E., Russi I., Grillotti E., Manzocchi S., Simonato G., Beraldo P., Viglietti A. (2019). Occurrence of canine and feline extra-intestinal nematodes in key endemic regions of Italy. Acta Trop..

[42] De Zan G., Citterio C.V., Danesi P., Gaspardis G., Gabassi E., Panciera L., Zanardello C., Binato G., Cocchi M. (2021). Angiostrongylosis in northeastern Italy: First report of two autochthonous fatal cases in dogs and first detection in a wild red fox. Vet. Parasitol. Reg. Stud. Rep..

[43] Magi M., Guardone L., Dell’omodarme M., Prati M., Mignone W., Torracca B., Monni G., Macchioni F. (2010). *Angiostrongylus vasorum* in red foxes (*Vulpes vulpes*) and badgers (*Meles meles*) from Central and Northern Italy. Hystrix Ital. J. Mammal..

[44] Di Cesare A., Traversa D. (2014). Canine angiostrongylosis: Recent advances in diagnosis, prevention, and treatment. Vet. Med..

[45] Del Prete L., Maurelli M.P., Pennacchio S., Bosco A., Musella V., Ciuca L., Cringoli G., Rinaldi L. (2015). *Dirofilaria immitis* and *Angiostrongylus vasorum*: The contemporaneous detection in kennels. BMC Vet. Res..

[46] Olivieri E., Zanzani S.A., Gazzonis A.L., Giudice C., Brambilla P., Alberti I., Romussi S., Lombardo R., Mortellaro C.M., Banco B. (2017). *Angiostrongylus vasorum* infection in dogs from a cardiopulmonary dirofilariosis endemic area of Northwestern Italy: A case study and a retrospective data analysis. BMC Vet. Res..

[47] Guardone L., Schnyder M., Macchioni F., Deplazes P., Magi M. (2013). Serological detection of circulating *Angiostrongylus vasorum* antigen and specific antibodies in dogs from central and northern Italy. Vet. Parasitol..

[48] Boitani L., Lovari S., Vigna Taglianti A. (2003). Fauna d’Italia, Mammalia III: Carnivora—Artiodactyla.

[49] Traversa D., Morelli S., Di Cesare A., Diakou A. (2021). Felid cardiopulmonary nematodes: Dilemmas solved and new questions posed. Pathogens.

[50] Miterpáková M., Schnyder M., Schaper R., Hurníková Z., Cabanova V. (2015). Serological survey for canine angiostrongylosis in Slovakia. Helminthologia.

[51] Schnyder M., Stebler K., Naucke T.J., Lorentz S., Deplazes P. (2014). Evaluation of a rapid device for serological in-clinic diagnosis of canine angiostrongylosis. Parasites Vectors.

[52] Schnyder M., Jefferies R., Schucan A., Morgan E.R., Deplazes P. (2015). Comparison of coprological, immunological and molecular methods for the detection of dogs infected with *Angiostrongylus vasorum* before and after anthelmintic treatment. Parasitology.

[53] Cury M.C., Lima W.S., Vitor R.W.A. (1996). Enzyme-Linked Immunosorbent Assay (ELISA) for the diagnosis of *Angiostrongylus vasorum* (Baillet, 1866) infection in dogs. Rev. Méd. Vét..

[54] Schucan A., Schnyder M., Tanner I., Barutzki D., Traversa D., Deplazes P. (2012). Detection of specific antibodies in dogs infected with *Angiostrongylus vasorum*. Vet. Parasitol..

[55] Signorini M., Cassini R., Drigo M., Frangipane di Regalbono A., Pietrobelli M., Montarsi F., Stensgaard A.S. (2014). Ecological niche model of *Phlebotomus perniciosus*, the main vector of canine leishmaniasis in north-eastern Italy. Geospat. Health.

[56] Mendoza-Roldan J., Benelli G., Panarese R., Iatta R., Furlanello T., Beugnet F., Zatelli A., Otranto D. (2020). *Leishmania infantum* and *Dirofilaria immitis* infections in Italy, 2009–2019: Changing distribution patterns. Parasites Vectors.

[57] De Massis F., Ippoliti C., Simona I., Tittarelli M., Pelini S., Giansante D., Ciarrocchi A. (2020). Canine leishmaniasis: Serological results in private and kennel dogs tested over a six-year period (2009–2014) in Abruzzo and Molise regions, Italy. Microorganisms.

[58] Biglino A., Bolla C., Concialdi E., Trisciuoglio A., Romano A., Ferroglio E. (2010). Asymptomatic *Leishmania infantum* infection in an area of northwestern Italy (Piedmont region) where such infections are traditionally nonendemic. J. Clin. Microbiol..

[59] Cassini R., Signorini M., Frangipane di Regalbono A., Natale A., Montarsi F., Zanaica M., Brichese M., Simonato G., Borgato S., Babiker A. (2013). Preliminary study of the effects of preventive measures on the prevalence of canine leishmaniosis in a recently established focus in northern Italy. Vet. Ital..

[60] Brianti E., Panarese R., Napoli E., De Benedetto G., Gaglio G., Bezerra-Santos M.A., Mendoza-Roldan J.A., Otranto D. (2021). *Dirofilaria immitis* infection in the Pelagie archipelago: The southernmost hyperendemic focus in Europe. Transbound Emerg. Dis..

[61] Gradoni L., Ferroglio E., Zanet S., Mignone W., Venco L., Bongiorno G., Fiorentino E., Cassini R., Grillini M., Simonato G. Monitoring and detection of new endemic foci of canine leishmaniosis in northern continental Italy: An update from a study involving five regions (2018–2019). Proceedings of the XXXIII Conference of the Italian Society of Parasitology/2021 ESDA EVENT.

[62] Olivieri E., Zanzani S.A., Latrofa M.S., Lia R.P., Dantas-Torres F., Otranto D., Manfredi M.T. (2016). The southernmost foci of *Dermacentor reticulatus* in Italy and associated *Babesia canis* infection in dogs. Parasites Vectors.

[63] Traversa D., Di Cesare A., Simonato G., Cassini R., Merola C., Diakou A., Halos L., Beugnet F., Frangipane di Regalbono A. (2017). Zoonotic intestinal parasites and vector-borne pathogens in Italian shelter and kennel dogs. Comp. Immunol. Microbiol. Infect. Dis..

[64] Socolovschi C., Gaudart J., Bitam I., Huynh T.P., Raoult D., Parola P. (2012). Why are there so few *Rickettsia conorii*-infected *Rhipicephalus sanguineus* ticks in the wild?. PLoS Negl. Trop. Dis..

[65] Maurelli M.P., Pepe P., Colombo L., Armstrong R., Battisti E., Morgoglione M.E., Counturis D., Rinaldi L., Cringoli G., Ferroglio E. (2018). A national survey of Ixodidae ticks on privately owned dogs in Italy. Parasites Vectors.

[66] Snellgrove A.N., Krapiunaya I., Ford S.L., Stanley H.M., Wickson A.G., Hartzer K.L., Levin M.L. (2020). Vector competence of *Rhipicephalus sanguineus* sensu stricto for *Anaplasma platys*. Ticks Tick-Borne Dis..

[67] Rubel F., Brugger K., Pfeffer M., Chitimia-Dobler L., Didyk Y.M., Leverenz S., Dautel H., Kahl O. (2016). Geographical distribution of *Dermacentor marginatus* and *Dermacentor reticulatus* in Europe. Ticks Tick-Borne Dis..

[68] Yamane I., Thomford J.W., Gardner I.A., Dubey J.P., Levy M., Conrad P.A. (1993). Evaluation of the indirect fluorescent antibody test for diagnosis of *Babesia gibsoni* infections in dogs. Am. J. Vet. Res..

[69] Solano-Gallego L., Trotta M., Carli E., Carcy B., Caldin M., Furlanello T. (2008). *Babesia canis canis* and *Babesia canis vogeli* clinicopathological findings and DNA detection by means of PCR-RFLP in blood from Italian dogs suspected of tick-borne disease. Vet. Parasitol..

[70] Pantchev N., Schnyder M., Vrhovec M.G., Schaper R., Tsachev I. (2015). Current surveys of the seroprevalence of Borrelia burgdorferi, Ehrlichia canis, Anaplasma phagocytophilum, Leishmania infantum, Babesia canis, Angiostrongylus vasorum and Dirofilaria immitis in Dogs in Bulgaria. Parasitol. Res..

[71] Iliev P.T., Kirkova Z.T., Tonev A.S. (2020). Preliminary study on the prevalence of endoparasite infections and vector-borne diseases in outdoor dogs in Bulgaria. Helminthologia.

[72] Manev I. (2020). Serological survey of vector-borne pathogens in stray dogs from Sofia area, Bulgaria. Vet. Parasitol. Reg. Stud. Rep..

[73] Pejchalová K., Zákovská A., Fucík K., Schánilec P. (2006). Serological confirmation of *Borrelia burgdorferi* infection in dogs in the Czech Republic. Vet. Res. Commun..

[74] Kybicová K., Schánilec P., Hulínská D., Uherková L., Kurzová Z., Spejchalová S. (2009). Detection of *Anaplasma phagocytophilum* and *Borrelia burgdorferi* sensu lato in dogs in the Czech Republic. Vector Borne Zoonotic Dis..

[75] Hajnalová M., Svobodová V., Schnyder M., Schaper R., Svoboda M. (2017). Faecal detection of the lungworms *Crenosoma vulpis* and *Angiostrongylus vasorum* and serological detection of *A. vasorum* in dogs from the Czech Republic. Acta Vet. Brno.

[76] Al-Sabi M.N.S., Deplazes P., Webster P., Willesen J.L., Davidson R.K., Kapel C.M.O. (2010). PCR detection of *Angiostrongylus vasorum* in faecal samples of dogs and foxes. Parasitol. Res..

[77] Pérez Vera C., Kapiainen S., Junnikkala S., Aaltonen K., Spillmann T., Vapalahti O. (2014). Survey of selected tick-borne diseases in dogs in Finland. Parasites Vectors.

[78] Tiškina V., Lindqvist E.L., Blomqvist A.C., Orav M., Stensvold C.R., Jokelainen P. (2019). Autochthonous *Angiostrongylus vasorum* in Finland. Vet. Rec. Open.

[79] Pantchev N., Schaper R., Limousin S., Norden N., Weise M., Lorentzen L. (2009). Occurrence of *Dirofilaria immitis* and tick-borne infections caused by *Anaplasma phagocytophilum*, *Borrelia burgdorferi* sensu lato and *Ehrlichia canis* in domestic dogs in France: Results of a countrywide serologic survey. Parasitol. Res..

[80] Schnyder M., Bilbrough G., Hafner C., Schaper R. (2017). *Angiostrongylus vasorum*, “The French Heartworm”: A serological survey in dogs from France introduced by a brief historical review. Parasitol. Res..

[81] Pantchev N., Norden N., Lorentzen L., Rossi M., Rossi U., Brand B., Dyachenko V. (2009). Current surveys on the prevalence and distribution of *Dirofilaria* spp. in dogs in Germany. Parasitol. Res..

[82] Barutzki D., Schaper R. (2009). Natural infections of *Angiostrongylus vasorum* and *Crenosoma vulpis* in dogs in Germany (2007–2009). Parasitol. Res..

[83] Barutzki D., Schaper R. (2011). Results of parasitological examinations of faecal samples from cats and dogs in Germany between 2003 and 2010. Parasitol. Res..

[84] Schulz B.S., Seybold N., Sauter-Louis C., Hartmann K. (2013). Prevalence of *Angiostrongylus vasorum* and *Crenosoma vulpis* in dogs in Bavaria. Berl. Münch. Tierärztl. Wochenschr..

[85] Liesner J.M., Krücken J., Schaper R., Pachnicke S., Kohn B., Müller E., Schulze C., von Samson-Himmelstjerna G. (2016). Vector-borne pathogens in dogs and red foxes from the federal state of Brandenburg, Germany. Vet. Parasitol..

[86] Vrhovec M.G., Pantchev N., Failing K., Bauer C., Travers-Martin N., Zahner H. (2017). Retrospective analysis of canine vector-borne diseases (CVBD) in germany with emphasis on the endemicity and risk factors of Leishmaniosis. Parasitol. Res..

[87] Schäfer I., Volkmann M., Beelitz P., Merle R., Müller E., Kohn B. (2019). Retrospective analysis of vector-borne infections in dogs after travelling to endemic areas (2007–2018). Vet. Parasitol. X.

[88] Papazahariadou M., Founta A., Papadopoulos E., Chliounakis S., Antoniadou-Sotiriadou K., Theodorides Y. (2007). Gastrointestinal parasites of shepherd and hunting dogs in the Serres Prefecture, Northern Greece. Vet. Parasitol..

[89] Angelou A., Gelasakis A.I., Verde N., Pantchev N., Schaper R., Chandrashekar R., Papadopoulos E. (2019). Prevalence and risk factors for selected canine vector-borne diseases in Greece. Parasites Vectors.

[90] Angelou A., Gelasakis A.I., Schnyder M., Schaper R., Papadopoulos E. (2020). The “French heartworm” in Greece: A countrywide serological survey of *Angiostrongylus vasorum* infection by combined detection of circulating antigens and specific antibodies. Vet. Parasitol. Reg. Stud. Rep..

[91] Hofmann M., Hodžić A., Pouliou N., Joachim A. (2019). Vector-borne pathogens affecting shelter dogs in eastern Crete, Greece. Parasitol. Res..

[92] Kostopoulou D., Gizzarelli M., Ligda P., Foglia Manzillo V., Saratsi K., Montagnaro S., Shunack B., Boegel A., Pollmeier A., Oliva G. (2020). Mapping the canine vector-borne disease risk in a Mediterranean area. Parasites Vectors.

[93] Hamel D., Silaghi C., Lescai D., Pfister K. (2012). Epidemiological aspects on vector-borne infections in stray and pet dogs from Romania and Hungary with focus on *Babesia* spp.. Parasitol. Res..

[94] Farkas R., Gyurkovszky M., Lukács Z., Aladics B., Solymosi N. (2014). Seroprevalence of some vector-borne infections of dogs in Hungary. Vector Borne Zoonotic Dis..

[95] Schnyder M., Schaper R., Lukács Z., Hornok S., Farkas R. (2015). Combined serological detection of circulating *Angiostrongylus vasorum* antigen and parasite-specific antibodies in dogs from Hungary. Parasitol. Res..

[96] Garcia-Campos A., Power C., O’Shaughnessy J., Browne C., Lawlor A., McCarthy G., O’Neill E.J., de Waal T. (2019). One-year parasitological screening of stray dogs and cats in County Dublin, Ireland. Parasitology.

[97] Di Cesare A., Castagna G., Meloni S., Milillo P., Latrofa S., Otranto D., Traversa D. (2011). Canine and feline infections by cardiopulmonary nematodes in central and southern Italy. Parasitol. Res..

[98] Pennisi M.G., Caprì A., Solano-Gallego L., Lombardo G., Torina A., Masucci M. (2012). Prevalence of antibodies against *Rickettsia conorii*, *Babesia canis*, *Ehrlichia canis*, and *Anaplasma phagocytophilum* antigens in dogs from the Stretto di Messina area (Italy). Ticks Tick-Borne Dis..

[99] Riggio F., Mannella R., Ariti G., Perrucci S. (2013). Intestinal and lung parasites in owned dogs and cats from central Italy. Vet. Parasitol..

[100] Pipia A.P., Varcasia A., Tosciri G., Seu S., Manunta M.L., Mura M.C., Sanna G., Tamponi C., Brianti E., Scala A. (2014). New insights onto cardiopulmonary nematodes of dogs in Sardinia, Italy. Parasitol. Res..

[101] Rinaldi L., Cortese L., Meomartino L., Pagano T.B., Pepe P., Cringoli G., Papparella S. (2014). *Angiostrongylus vasorum*: Epidemiological, clinical and histopathological insights. BMC Vet. Res..

[102] Otranto D., Dantas-Torres F., Mihalca A., Traub R., Lappin M., Baneth G. (2017). Zoonotic parasites of sheltered and stray dogs in the era of the global economic and political crisis. Trends Parasitol..

[103] Piantedosi D., Neola B., D’Alessio N., Di Prisco F., Santoro M., Pacifico L., Sgroi G., Auletta L., Buch J., Chandrashekar R. (2017). Seroprevalence and risk factors associated with *Ehrlichia canis*, *Anaplasma* spp.; *Borrelia burgdorferi* sensu lato, and *D. immitis* in hunting dogs from southern Italy. Parasitol. Res..

[104] Foglia Manzillo V., Gizzarelli M., Vitale F., Montagnaro S., Torina A., Sotera S., Oliva G. (2018). Serological and entomological survey of canine leishmaniasis in Lampedusa island, Italy. BMC Vet. Res..

[105] De Liberato C., Berrilli F., Odorizi L., Scarcella R., Barni M., Amoruso C., Scarito A., Filippo M.M.D., Carvelli A., Iacoponi F. (2018). Parasites in stray dogs from Italy: Prevalence, risk factors and management concerns. Acta Parasitol..

[106] Sauda F., Malandrucco L., Macrì G., Scarpulla M., De Liberato C., Terracciano G., Fichi G., Berrilli F., Perrucci S. (2018). *Leishmania infantum*, *Dirofilaria* spp. and other endoparasite infections in kennel dogs in central Italy. Parasite.

[107] Schnyder M., Schaper R., Pantchev N., Kowalska D., Szwedko A., Deplazes P. (2013). Serological detection of circulating *Angiostrongylus vasorum* antigen- and parasite-specific antibodies in dogs from Poland. Parasitol. Res..

[108] Krämer F., Schaper R., Schunack B., Połozowski A., Piekarska J., Szwedko A., Jodies R., Kowalska D., Schüpbach D., Pantchev N. (2014). Serological detection of *Anaplasma phagocytophilum*, *Borrelia burgdorferi* sensu lato and *Ehrlichia canis* antibodies and *Dirofilaria immitis* antigen in a countrywide survey in dogs in Poland. Parasitol. Res..

[109] Dzięgiel B., Adaszek Ł., Carbonero A., Łyp P., Winiarczyk M., Dębiak P., Winiarczyk S.L. (2016). Detection of canine vector-borne diseases in eastern Poland by ELISA and PCR. Parasitol. Res..

[110] Alexandre N., Santos A.S., Bacellar F., Boinas F.J., Núncio M.S., de Sousa R. (2011). Detection of *Rickettsia conorii* strains in Portuguese dogs (*Canis familiaris*). Ticks Tick-Borne Dis..

[111] Nabais J., Alho A.M., Gomes L., Ferreira da Silva J., Nunes T., Vicente G., Madeira de Carvalho L. (2014). *Aelurostrongylus abstrusus* in cats and *Angiostrongylus vasorum* in dogs from Lisbon, Portugal. Acta Parasitol. Port..

[112] Maia C., Altet L., Serrano L., Cristóvão J.M., Tabar M.D., Francino O., Cardoso L., Campino L., Roura X. (2016). Molecular detection of *Leishmania infantum*, filariae and *Wolbachia* spp. in dogs from southern Portugal. Parasites Vectors.

[113] Serrão I., São Braz B., Dargent Figueiredo M., Coimbra M., Brancal H., Fernandes M.C., Lopes A.P., Pimenta P., Martins A., Pereira A. (2016). Preliminary report on the prevalence of *Angiostrongylus vasorum* infection in dogs from Portugal adopting a commercially available test kit for serological analysis. Vet. Parasitol. Reg. Stud. Rep..

[114] Maia C., Cristóvão J.M., Pereira A., Parreira R., Campino L. (2019). Detection of *Rickettsia conorii israelensis* DNA in the blood of a cat and a dog from Southern Portugal. Top. Companion Anim. Med..

[115] Simin S., Spasojević Kosić L., Kuruca L., Pavlović I., Savović M., Lalošević V. (2014). Moving the boundaries to the South-East: First record of autochthonous *Angiostrongylus vasorum* infection in a dog in Vojvodina province, northern Serbia. Parasites Vectors.

[116] Savic S., Vidic B., Grgic Z., Petrovic T., Potkonjak A., Cupina A., Vaselek S., Petric D., Samie A. (2015). Dirofilariosis and leishmaniasis in the northern region of Serbia. An Overview of Tropical Diseases.

[117] Kovačević Filipović M.M., Beletić A.D., Ilić Božović A.V., Milanović Z., Tyrrell P., Buch J., Chandrashekar R. (2018). Molecular and Serological Prevalence of *Anaplasma phagocytophilum*, *A. platys*, *Ehrlichia canis*, *E. chaffeenses*, *E. ewingii*, *Borrelia burgdorferi*, *Babesia canis*, *B. gibsoni* and *B. vogeli* among clinically healthy outdoor dogs in Serbia. Vet. Parasitol. Reg. Stud. Rep..

[118] Hurníková Z., Miterpáková M., Mandelík R. (2013). First autochthonous case of canine *Angiostrongylus vasorum* in Slovakia. Parasitol. Res..

[119] Miterpáková M., Hurníková Z., Zalewski A.P. (2014). The first clinically manifested case of angiostrongylosis in a dog in Slovakia. Acta Parasitol..

[120] Čabanová V., Pantchev N., Hurníková Z., Miterpáková M. (2015). Recent study on canine vector-borne zoonoses in southern Slovakia—Serologic survey. Acta Parasitol..

[121] Solano-Gallego L., Llull J., Osso M., Hegarty B., Breitschwerdt E. (2006). A serological study of exposure to arthropod-borne pathogens in dogs from northeastern Spain. Vet. Res..

[122] Amusategui I., Tesouro M.A., Kakoma I., Sainz A. (2008). Serological reactivity to Ehrlichia canis, Anaplasma phagocytophilum, Neorickettsia risticii, Borrelia burgdorferi and Rickettsia conorii in dogs from northwestern Spain. Vector Borne Zoonotic Dis..

[123] Couto C.G., Lorentzen L., Beall M.J., Shields J., Bertolone N., Couto J.I., Couto K.M., Nash S., Slack J., Kvitko H. (2010). Serological study of selected vector-borne diseases in shelter dogs in central Spain using point-of-care assays. Vector Borne Zoonotic Dis..

[124] Miró G., Montoya A., Roura X., Gálvez R., Sainz A. (2013). Seropositivity rates for agents of canine vector-borne diseases in Spain: A multicentre study. Parasites Vectors.

[125] Montoya-Alonso J.A., Carretón E., Simón L., González-Miguel J., García-Guasch L., Morchón R., Simón F. (2015). Prevalence of *Dirofilaria immitis* in dogs from Barcelona: Validation of a geospatial prediction model. Vet. Parasitol..

[126] Espejo E., Andrés M., Pérez J., Prat J., Guerrero C., Muñoz M.T., Alegre M.D., Lite J., Bella F. (2016). Prevalence of antibodies to *Rickettsia conorii* in human beings and dogs from Catalonia: A 20-year perspective. Epidemiol. Infect..

[127] Montoya-Alonso J.A., Morchón R., Falcón-Cordón Y., Falcón-Cordón S., Simón F., Carretón E. (2017). Prevalence of heartworm in dogs and cats of Madrid, Spain. Parasites Vectors.

[128] Velez R., Ballart C., Domenech E., Abras A., Fernández-Arévalo A., Gómez S.A., Tebar S., Muñoz C., Cairó J., Gállego M. (2019). Seroprevalence of canine *Leishmania infantum* infection in the Mediterranean region and identification of risk factors: The example of North-Eastern and Pyrenean areas of Spain. Prev. Vet. Med..

[129] Díaz-Regañón D., Roura X., Suárez M.L., León M., Sainz Á. (2020). Serological evaluation of selected vector-borne pathogens in owned dogs from northern Spain based on a multicenter study using a commercial test. Parasites Vectors.

[130] Montoya-Alonso J.A., Morchón R., Costa-Rodríguez N., Matos J.I., Falcón-Cordón Y., Carretón E. (2020). Current distribution of seleced Vector-Borne Diseases in dogs in Spain. Front. Vet. Sci..

[131] Morchón R., Montoya-Alonso J.A., Sánchez-Agudo J.Á., de Vicente-Bengochea J., Murcia-Martínez X., Carretón E. (2021). Angiostrongylus vasorum in domestic dogs in castilla y León, Iberian Peninsula, Spain. Animals.

[132] Cavalera M.A., Schnyder M., Gueldner E.K., Furlanello T., Iatta R., Brianti E., Strube C., Colella V., Otranto D. (2019). Serological survey and risk factors of *Aelurostrongylus abstrusus* infection among owned cats in italy. Parasitol. Res..

[133] Gueldner E.K., Gilli U., Strube C., Schnyder M. (2019). Seroprevalence, biogeographic distribution and risk factors for *Aelurostrongylus abstrusus* infections in Swiss cats. Vet. Parasitol..

[134] Di Cesare A., Gueldner E.K., Traversa D., Veronesi F., Morelli S., Crisi P.E., Pampurini F., Strube C., Schnyder M. (2019). Seroprevalence of antibodies against the cat lungworm *Aelurostrongylus abstrusus* in cats from endemic areas of Italy. Vet. Parasitol..

[135] Morelli S., Diakou A., Di Cesare A., Schnyder M., Colombo M., Strube C., Dimzas D., Latino R., Traversa D. (2020). Feline lungworms in Greece: Copromicroscopic, molecular and serological study. Parasitol. Res..

[136] Genchi C., Kramer L.H. (2020). The prevalence of *Dirofilaria immitis* and *D. repens* in the Old World. Vet. Parasitol..

[137] Gianchecchi E., Montomoli E. (2020). The enemy at home: Leishmaniasis in the Mediterranean basin, Italy on the focus. Expert Rev. Anti-Infect. Ther..

[138] Lebon W., Tielemans E., Rehbein S., Dumont P., Yoon S., Beugnet F., Jeannin P., Larsen D., Halos L. (2016). Monthly administrations of milbemycin oxime plus afoxolaner chewable tablets to prevent *Angiostrongylus vasorum* infection in dogs. Parasites Vectors.

[139] Böhm C., Petry G., Schmidt H., Raue K., Barthel F., Schaper R. (2017). Evaluation of the persistent preventive efficacy of 2.5 % moxidectin/10% imidacloprid spot-on (Advocate^®^, Advantage^®^ Multi) in dogs experimentally infected with *Angiostrongylus vasorum*. Parasitol. Res..

[140] Becskei C., Willesen J.L., Schnyder M., Wozniakiewicz M., Miroshnikova N., Mahabir S.P. (2020). Field safety and efficacy of an orally administered combination of sarolaner, moxidectin and pyrantel (Simparica Trio^®^) for the prevention of angiostrongylosis in dogs presented as veterinary patients. Parasites Vectors.

[141] Noack S., Harrington J., Carithers D.S., Kaminsky R., Selzer P.M. (2021). Heartworm disease—Overview, intervention, and industry perspective. Int. J. Parasitol. Drugs Drug Resist..

[142] Bowman D.D. (2012). Heartworms, macrocyclic lactones, and the specter of resistance to prevention in the United States. Parasites Vectors.

[143] Diakou A. Concern for Dirofilaria immitis and LOE/Resistance: Current situation in the USA and Europe, and future scenarios. Proceedings of the XXXIII Conference of the Italian Society of Parasitology/2021 ESDA EVENT.

[144] Schorderet-Weber S., Noack S., Selzer P.M., Kaminsky R. (2017). Blocking transmission of vector-borne diseases. Int. J. Parasitol. Drugs Drug Resist..

[145] Mencke N. (2006). Acaricidal and repellent properties of permethrin, its role in reducing transmission of vector-borne pathogens. Parassitologia.

[146] Ferroglio E., Poggi M., Trisciuoglio A. (2008). Evaluation of 65% permethrin spot-on and deltamethrin-impregnated collars for canine *Leishmania infantum* infection prevention. Zoonoses Public Health.

[147] Brianti E., Gaglio G., Napoli E., Falsone L., Prudente C., Basano F.S., Latrofa M.S., Tarallo V.D., Dantas-Torres F., Capelli G. (2014). Efficacy of a slow-release imidacloprid (10%)/flumethrin (4.5%) collar for the prevention of canine leishmaniosis. Parasites Vectors.

[148] Jongejan F., Crafford D., Erasmus H., Fourie J.J., Schunack B. (2016). Comparative efficacy of oral administrated afoxolaner (NexGard™) and fluralaner (Bravecto™) with topically applied permethrin/imidacloprid (Advantix(^®^)) against transmission of *Ehrlichia canis* by infected *Rhipicephalus sanguineus* ticks to dogs. Parasites Vectors.

[149] Miglianico M., Eldering M., Slater H., Ferguson N., Ambrose P., Lees R.S., Koolen K.M.J., Pruzinova K., Jancarova M., Volf P. (2018). Repurposing isoxazoline veterinary drugs for control of vector-borne human diseases. Proc. Natl. Acad. Sci. USA.

[150] Queiroga T.B.D., Ferreira H.R.P., Dos Santos W.V., de Assis A.B.L., de Araújo Neto V.T., da Câmara A.C.J., Fagundes Nedo J.C., dos Reis R.K., Nascimento M.S.L., Gama R.A. (2020). Fluralaner (Bravecto^®^) induces long-term mortality of *Lutzomyia longipalpis* after a blood meal in treated dogs. Parasites Vectors.

[151] Panarese R., Iatta R., Mendoza-Roldan J.A., Zatelli A., Beugnet F., Otranto D. Insecticidal efficacy of afoxolaner (NexGard^®^) in the prevention of *Leishmania infantum* and *Dirofilaria immitis* transmission to sheltered dogs in a high endemic area. Proceedings of the XXXIII Conference of the Italian Society of Parasitology/2021 ESDA EVENT.

[152] Schnyder M., Tanner I., Webster P., Barutzki D., Deplazes P. (2011). An ELISA for sensitive and specific detection of circulating antigen of *Angiostrongylus vasorum* in serum samples of naturally and experimentally infected dogs. Vet. Parasitol..

